# Lipid Peroxidation as the Mechanism Underlying Polycyclic Aromatic Hydrocarbons and Sunlight Synergistic Toxicity in Dermal Fibroblasts

**DOI:** 10.3390/ijms25031905

**Published:** 2024-02-05

**Authors:** Eloïse Larnac, Alicia Montoni, Valérie Haydont, Laurent Marrot, Patrick J. Rochette

**Affiliations:** 1 Centre de Recherche du CHU de Québec, Université Laval, Axe Médecine Régénératrice, Hôpital du Saint-Sacrement, Québec, QC G1S 4L8, Canada; eloise.larnac.1@ulaval.ca (E.L.); alicia.montoni@crchudequebec.ulaval.ca (A.M.); 2 Centre de Recherche en Organogénèse Expérimentale, Université Laval/LOEX, Québec, QC G1V 0A6, Canada; 3 Advanced Research, L’OREAL Research & Innovation, 93600 Aulnay-Sous-Bois, France; valerie.haydont@loreal.com (V.H.); laurent.marrot@loreal.com (L.M.); 4 Département d’Ophtalmologie et ORL-Chirurgie Cervico-Faciale, Faculté de Médecine, Université Laval, Québec, QC G1V 0A6, Canada

**Keywords:** ultraviolet A (UVA), Benzo[a]pyrene (BaP), lipid peroxidation, reactive oxygen species (ROS), skin dermal fibroblasts

## Abstract

Light and atmospheric pollution are both independently implicated in cancer induction and premature aging. Evidence has been growing more recently on the toxic synergy between light and pollutants. Polycyclic aromatic hydrocarbons (PAHs) originate from the incomplete combustion of organic matter. Some PAHs, such as the Benzo[a]pyrene (BaP), absorb ultraviolet A (UVA) wavelengths and can act as exogenous chromophores, leading to synergistic toxicity through DNA damage and cytotoxicity concomitant to ROS formation. In this study, we shed light on the mechanism underlying the toxic synergy between PAHs and UVA. Using dermal fibroblasts co-exposed to UVA and BaP, we have demonstrated that the photosensitization reaction causes mortality, which is most likely caused by ROS accumulation. We have shown that these ROS are concentrated in the lipids, which causes an important induction of lipid peroxidation and malondialdehyde, by-products of lipid peroxidation. We have also shown the accumulation of bulky DNA damage, most likely generated by these by-products of lipid peroxidation. To our knowledge, this study represents the first one depicting the molecular effects of photo-pollution on dermal skin.

## 1. Introduction

Skin is continuously coping with environmental stressors such as light exposure and atmospheric pollution. The toxicity of these environmental factors for skin cells and their implication in cancer induction or premature aging has been studied individually [1,2,3,4,5]. However, evidence is growing on the toxic interaction between light and pollutants [6,7,8,9,10]. This synergy most likely occurs through a photosensitization reaction [11,12], but the underlying mechanism remains largely unknown.

Within solar ultraviolet (UV) light, UVB and UVA represent 5–10% and 90–95% of the solar UV spectrum, respectively. It is well accepted that UVB are the solar rays involved in cutaneous cancers through the induction of pyrimidine dimers, the most abundant and mutagenic UV-induced DNA damage [13,14,15,16]. Although UVA radiations have been shown to induce mainly non-mutagenic CPD (i.e., predominantly at T-containing dipyrimidines) [17,18,19], their toxicity is mainly exerted through chromophore photosensitization reactions [20,21,22,23,24]. The penetration of UV radiation in human skin is wavelength-dependent. UVB wavelengths penetrate poorly in the skin and are mostly deleterious for the epidermis. UVA penetrate into the deeper layers of the skin and can be absorbed by the dermal layer [25]. 

Pollutants include a wide variety of toxic suspension particles. These particulate matters originate from the incomplete combustion of organic matter such as from cigarette smoke, coal combustion, or diesel motors [26]. Particulate matters contain many components, such as polycyclic aromatic hydrocarbons (PAHs), lipophilic compounds constituted of condensate benzene rings. Benzo[a]pyrene (BaP) is amongst the most abundant PAHs [27,28,29,30,31]. BaP has been recognized by the International Agency for Research on Cancer (IARC) as a confirmed carcinogen [28,29]. Due to the particle size, particulate matters translocate from the pulmonary alveoli to capillaries [26,32,33]. Then, PAHs can reach the dermis by blood circulation, where it they have been found in the nanomolar range [34,35]. At the epidemiological level, a correlation has been found between signs of skin aging and people living in polluted areas [1]. At the molecular level, exposure to cigarette smoke and diesel particulate extracts has been associated with an increase in MMP1 and MMP3 and a decrease in collagen neo-synthesis [36,37]. Human dermal fibroblasts exposed to particulate matters showed a decrease in cell viability, and an increase in MMP-1 and MMP-3 associated with a decrease in pro-collagen, TGF-β and elastin gene expression [38].

Some PAHs can act as exogenous chromophores [39]. We, and other groups, have previously showed [6,8] that PAHs absorb UVA wavelengths, leading to synergistic toxicity. This indicates that light exposure and pollution could synergize their effect, in the concept of photo-pollution [33,40]. It has been shown that the co-exposure to cigarette smoke and sunlight favours the formation of facial wrinkles and increase MMP1 expression [41,42,43]. Co-exposure to PAHs and UVA generate DNA damage and cytotoxicity concomitant to ROS formation [6,7,9,44,45,46,47]. PAHs in nM concentration, when exposed to UVA, lead to important photo-toxicity activity with an effect on keratinocyte proliferation, oxidative stress, and ATP production [8].

In the present study, we have gained insight into the molecular mechanisms involved in the toxic effect of photo-pollution on the skin. Indeed, we have demonstrated that a photosensitization reaction between UVA and BaP causes mortality, ROS accumulation, and lipid peroxidation. We have also shown the accumulation of bulky DNA damage, most likely generated by by-products of lipid peroxidation. To our knowledge, this study represents the first one depicting the molecular effects of photo-pollution on dermal skin. 

## 2. Results

### 2.1. Sensitivity to UVAssl- or VLssl-Induced Cell Death in BaP-Treated Fibroblasts

**Figure 1:**
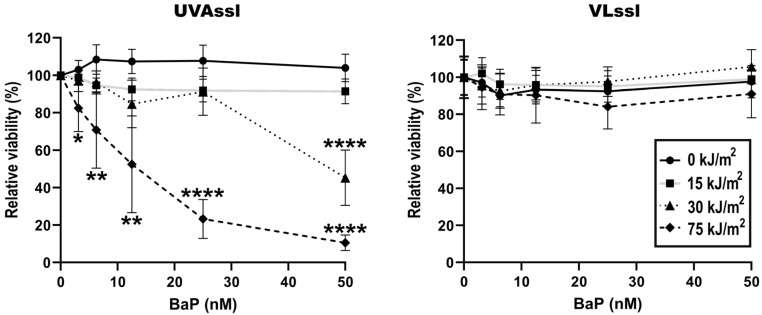
Sensitivity to UVAssl- or VLssl-induced cell death in BaP-treated fibroblasts. The viability of fibroblasts exposed to increasing BaP and UVAssl (**left**) or VLssl (**right**) was measured. Dermal fibroblasts were pre-treated with BaP in PBS for 30 min and exposed to light. Cell viability was assessed using an MTS assay that measured metabolic activity 24 h after the end of the exposure. Cells exposed to BaP are sensitive to UVAssl in a dose/concentration manner, but not to VLssl. Error bars are SD from four independent experiments. Differences between each condition and the control (0 kJ/m^2^) were assessed using one-way analysis of variance (ANOVA1) with the Tukey HSD procedure as a post hoc test (* *p* < 0.05, ** *p* < 0.005, **** *p* < 0.0001).

Dermal fibroblasts were treated with BaP and then irradiated with UVA or Visible Light (VL) using a solar simulator (SSL) with appropriate blocking filters. Cell viability was then determined using an MTS assay, 24 h post-exposure (Figure 1). Exposure to UVAssl and VLssl did not significantly affect fibroblasts’ viability up to 75 kJ/m^2^. When combined with BaP, 30 and 75 kJ/m^2^ of UVAssl induced a significant reduction in cell viability. More precisely, using 30 kJ/m^2^ of UVAssl, a significant viability decline was observable with 50 nM of BaP. When UVAssl irradiation was increased to 75 kJ/m^2^, as little as 3 nM BaP was sufficient to induce significant cell death. Combined with VLssl, BaP did not sensitize cells. These results show that BaP sensitized fibroblasts to UVAssl but not VLssl. This correlates with the fact that BaP absorbs only in the UVA wavelengths and not in the visible light spectrum. 

### 2.2. Induction of Oxidative Stress in BaP-Treated Fibroblasts Exposed to UVAssl or VLssl

**Figure 2:**
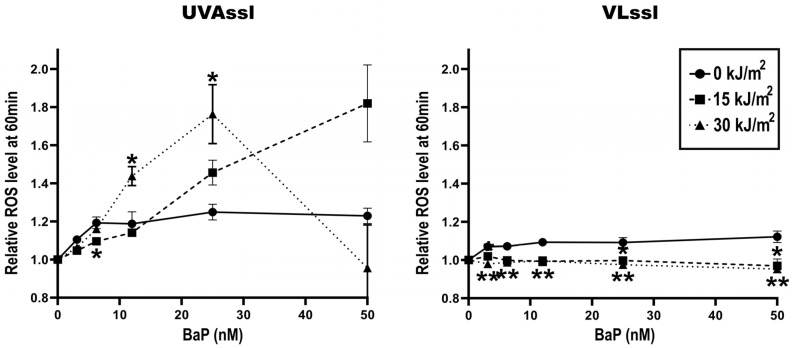
ROS induction by UVAssl or VLssl in BaP-treated fibroblasts. Oxidation-sensitive CM-H_2_DCFDA dye was used to measure the oxidation generated following UVAssl/VLssl and BaP co-exposure. Fibroblasts were pre-treated with BaP in PBS for 30 min and then exposed to UVAssl (**left**) or VLssl (**right**). CM-H_2_DCFDA was then incubated in cells for 60 min and the signal measured. We found an accumulation of ROS, dose- and concentration-dependent, in BaP/UVAssl-exposed fibroblasts (**left panel**). The important reduction in ROS observed at 50 nM BaP combined to 30 kJ/m^2^ UVAssl was most likely due to the decrease in metabolic/mitochondrial activity of the cells. In Bap/VLssl-exposed cells, we did not find any significant accumulation of ROS. Error bars are SD from four independent experiments. For each BaP concentration, differences between each condition and the control (0 kJ/m^2^) were assessed using one-way analysis of variance (ANOVA1) with the Tukey HSD procedure as a post hoc test (* *p* < 0.05, ** *p* < 0.005).

ROS formation, in BaP-treated (0–50 nM) fibroblasts exposed to UVAssl (0–30 kJ/m^2^) or VLssl (0–200 kJ/m^2^), was determined using the oxidant-sensitive indicator CM-H_2_DCFDA. Oxidation was measured 60 min following exposure, as previously performed [6]. BaP did not lead to ROS formation (Figure 2). We found no ROS formation when BaP pre-treated fibroblasts were exposed to VLssl. UVAssl-irradiated fibroblasts showed a formation of ROS which was BaP-concentration-dependent. Cells exposed to 12.5 or 25 nM BaP and 30 kJ/m^2^ had a significant increase in ROS formation compared to the unirradiated condition (+8% (*p* < 0.05) and +58% (*p* < 0.05), respectively). We suspect that the important reduction in ROS observed at 50 nM BaP combined to 30 kJ/m^2^ UVAssl was caused by a transitory decrease in the metabolic/mitochondrial activity of the cells. 

### 2.3. Lipid Peroxidation and MDA Formation in BaP/UVAssl-Treated Dermal Fibroblasts

**Figure 3:**
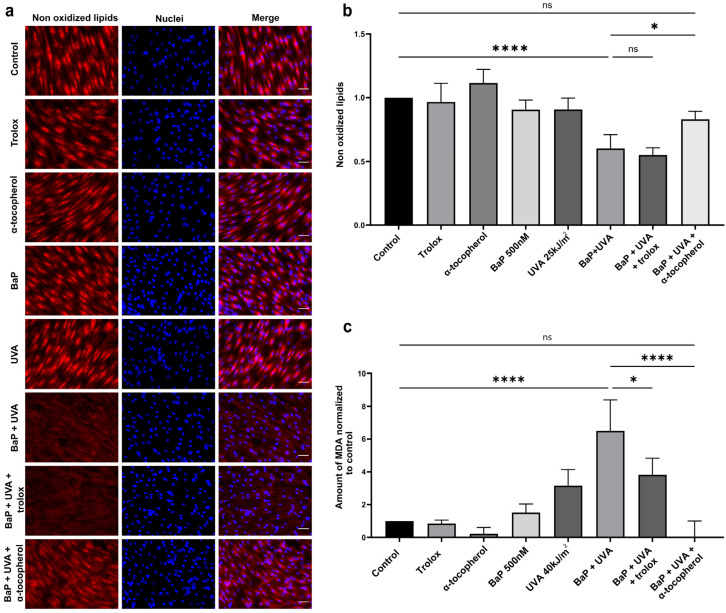
Lipid peroxidation in UVAssl and BaP co-exposure in fibroblasts. (**a**) Lipid peroxidation was assessed in fibroblasts using a lipophilic fluorescent dye sensitive to oxidation (red) and the nuclei were counterstained with Hoechst (blue). Cells with or without antioxidant pre-treatment were incubated with BaP (500 nM) in PBS for 30 min and then exposed to 25 kJ/m^2^ UVAssl. Two hours post-exposure, lipid peroxidation was assessed by fluorescent microscopy (scale bar = 50 µm). (**b**) Fluorescence signal quantification showed that co-exposure to BaP and UVAssl led to lipid peroxidation formation in skin fibroblasts. BaP/UVAssl-induced lipid peroxidation was significantly prevented by α-tocopherol but not with trolox. (**c**) MDA formation was measured in fibroblasts, with or without antioxidant pre-treatment. Cells were incubated with BaP and then exposed to 40 kJ/m^2^ UVAssl and MDA formation was assessed 2 h post-exposure. Co-exposure to BaP and UVAssl led to MDA formation in skin fibroblasts, whereas BaP or UVAssl alone did not have this effect. Cell pre-treatment with α-tocopherol decreased BaP/UVAssl-induced MDA formation more significatively than trolox. Error bars were SD from at least three independent experiments. Differences between each condition and the control were assessed using one-way analysis of variance (ANOVA1) with a Tukey HSD procedure as the post hoc test (ns: non significative difference * *p* < 0.05, **** *p* < 0.0001).

Since a significant level of ROS was only observed in UVAssl- and BaP-treated cells (Figure 2), we assessed lipid peroxidation in this condition. Lipid peroxidation was analysed using a fluorescent dye sensitive to oxidation. This dye loses its fluorescence when it interacts with peroxyl radicals, and we thus measured the loss of fluorescence as an indicator of peroxidation. BaP (500 nM) or UVAssl (25 kJ/m^2^) alone do not induce a measurable level of lipid peroxidation (Figure 3a,b). However, when combined, UVAssl and BaP induce a significant increase in lipid oxidation (increased by 1.7-fold, *p* < 0.0001). We tested two antioxidants for their ability to prevent lipid peroxidation, i.e., the lipophilic α-tocopherol and its hydrophilic counterpart, trolox. UVAssl/BaP-induced lipid peroxidation was significantly prevented by α-tocopherol (decreased by 1.4-fold, *p* < 0.05), but not trolox.

We then measured the levels of MDA, a lipid peroxidation by-product, in UVAssl and/or BaP-treated fibroblasts. BaP alone (500 nM) or UVAssl (40 kJ/m^2^) did not lead to a significant level of MDA. Co-exposure with BaP and UVAssl led to a significant increase in MDA production by over 6.5 times the basal level (*p* < 0.0001), further confirming the formation of lipid peroxidation. Pre-treatment with 100 µM α-tocopherol completely abolished the induction of UVAssl/BaP-induced MDA formation (decreased by 99.7%, *p* < 0.0001). Pre-treatment with trolox prevented a fraction of BaP/UVAssl-induced MDA production (decreased by 41.3%, *p* < 0.05). 

### 2.4. Bulky DNA Adducts

**Figure 4:**
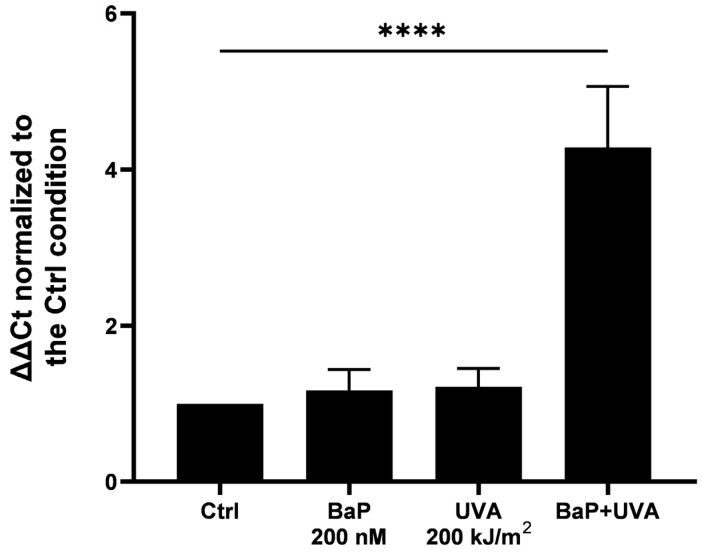
Bulky DNA adducts’ formation by UVAssl and BaP in fibroblasts. Bulky DNA adduct formation in UVAssl-exposed fibroblasts treated (or not) with BaP was measured. Dermal fibroblasts were pre-treated with BaP (200 nM) in PBS for 30 min and exposed to UVAssl (200 kJ/m^2^). After 6 h incubation, DNA was extracted and an LA-qPCR was performed. UVAssl- or BaP-exposed fibroblasts did not create bulky adducts, whereas bulky adducts were significantly induced in BaP/UVAssl-exposed fibroblasts. Error bars were SD from three independent experiments. Differences between each condition and the control were assessed using one-way analysis of variance (ANOVA1) with a Tukey HSD procedure as the post hoc test (**** *p* < 0.0001).

MDA can attack DNA to generate bulky DNA adducts [48,49]. We measured bulky DNA adduct formation upon BaP/UVAssl co-exposure using a novel qPCR technique we have adapted for this purpose. This qPCR technique is based on the reduction in amplification efficiency caused by bulky adducts. We compared the amplification efficiency of a long amplicon, where bulky adducts have a high probability of being found, with the efficiency of a very small amplicon. With this technique, we can determine the relative presence of bulky DNA damage. No measurable amount of bulky DNA adducts could be found in fibroblasts exposed only to BaP or UVAssl. However, fibroblasts co-exposed with BaP and UVAssl accumulated a measurable amount of bulky DNA adducts (increased by 4.3-fold, *p* < 0.0001—Figure 4).

## 3. Discussion

The synergistic effect between UV rays and pollutants (photo-pollution) is a subject of growing interest [7,9,40]. Most studies have described the combined effect of light and PAHs in the skin and eye [6,8,10], but the underlying molecular mechanisms are still poorly understood. In this study, we shed some light on the mechanisms involved in the toxicity of PAHs and UV light in dermal fibroblasts. We and others [6,8] have previously shown that BaP absorbs UVA wavelengths. The molecular effect of PAHs alone has been studied using concentration in the micromolar range [50,51]. It is difficult to precisely assess the quantity of PAHs that reache different human tissues, mainly because several parameters can influence their presence in the atmosphere. For example, PAHs are subjected to reactions of photooxidation and oxidation with the ozone, which can significantly lower their presence [52,53]. In this study, we chose PAHs concentrations in the range of nM since it corresponds to the concentrations found in the blood of people living in polluted environments or amongst smokers [34,54]. Irradiation doses used in this study represent less than 1 h of zenith sun exposure (approx. 55 kJ/m^2^ UVA) [55], except for the bulky DNA adduct formation experiment, where the UVA irradiation represents an exposure of 4 h of zenith sun.

Consistent with the absorption spectrum of BaP, exposure to UVAssl but not VLssl leads to a decrease in viability in cells exposed to BaP (Figure 1). Since the photo-toxicity of BaP is consistent with its absorption capacity, these results strongly suggest that photosensitivity results from light absorption. The wavelength specificity of the BaP has also been shown by Crallan et al. in keratinocytes [46]. These results shed light on the importance of filtering wavelengths over 340 nm in a complete solar protection strategy, especially for people exposed to polluted environments or cigarette smoke. 

Consistent with the mortality induced by UVAssl/BaP co-exposed cells, we found a significant amount of ROS when fibroblasts were treated in this condition. ROS induction is UVAssl-dose/BaP-concentration-dependent up to 30 kJ/m^2^ of UVAssl (Figure 2). We hypothesized that the decrease in ROS induction in the highest UVAssl/BaP exposure tested (Figure 2) is caused by metabolism/mitochondrial affectation, which is consistent with the viability loss at this dose/concentration (Figure 1). Typically, direct photosensitization reactions generate singlet oxygen through energy transfer (type II mechanism). This singlet oxygen can attack proteins, and guanine in DNA, but also lipids, and lead to cell death [22,23,24]. This ROS induction is not the result of direct photo-sensitization reactions since we could not measure any singlet oxygen induction following UVAssl and BaP co-exposure. The ROS measured (Figure 2) might come from the type I mechanism (electron transfer), inducing hydrogen peroxide as well as the highly reactive hydroxyl radical. These reactive ROS can attack lipids and proteins, but also DNA, to mainly induce 8-oxo-7,8-dihydroguanine [22,23]. The lipophilic nature of BaP suggests that lipid peroxidation is the main target of UVA/BaP-induced ROS. Nevertheless, it would have been relevant to study the damage induced in genomic and mitochondrial DNA in order to determine the real extent of the damage caused by UVA/BaP synergy. Taken together, these results indicate that the combination of UVAssl and BaP induces oxidative stress up to a point that it is eventually lethal for fibroblasts. It is important to note that this ROS induction and mortality occurs at a physiological UVAssl dose (<1 h zenith sun exposure) and with lower than 50 nM of BaP.

We then attempted to decipher the mechanisms underlying delayed ROS and mortality induced by UVAssl and BaP co-exposure. The lipophilic nature of BaP led us to investigate the formation of lipid peroxidation. Co-exposure to UVAssl/BaP induces lipid peroxidation in fibroblasts, as evidenced by the lipid-specific oxidation-sensitive probe and MDA formation (Figure 3). It is further evidenced by the fact that lipid peroxidation could be prevented by α-tocopherol (Figure 3). MDA, a well-accepted marker for lipid peroxidation, is an aldehyde that can attack DNA, leading to DNA damage [48,49]. More precisely, MDA can react with guanine to generate M1dG. M1dG is a bulky adduct known to lead to frameshift mutations and base pair substitutions [56]. Since we found a significant amount of MDA generated in fibroblasts co-exposed with UVAssl and BaP, we investigated the formation of bulky DNA adducts. Using a LA-qPCR technique we have adapted to allow the relative quantification of bulky DNA adducts, we have measured DNA damage induced by UVAssl/BaP. We have found an important formation of DNA adducts in fibroblasts co-exposed with UVAssl and BaP. The technique does not discriminate between DNA adducts as far as they halt DNA polymerase progression. They can result from other lipid peroxidation by-products than MDA, such as the 4-HNE [49]. DNA bulky adducts can also be the result of BaP metabolization by P450 cytochromes, which converts them into a reactive diol epoxide derivative, the BPDE, that can induce DNA bulky adducts [57]. They can also be the result of a direct structural photo-modification of the BaP into a still-unknown DNA-damaging product [58,59]. This result is consistent with the presence of the bulky DNA adducts we have found in retinal cells exposed to IcdP and blue light [60]. In the context of dermal aging, the accumulation of bulky adducts in fibroblasts can then lead to cellular damage and the premature senescence of the cells, impacting the functionality of the cells and their capacity to produce and renew the dermal extracellular matrix [4,61,62,63,64].

Our results show, for the first time, the effect of exposure of a major PAH to different wavelengths in dermal skin fibroblasts. The significant lipid peroxidation induced is concerning for the membrane stability of different organelles, including mitochondria, in which BaP has been shown to accumulate [65]. According to our results, good prevention strategies need to be emphasized in polluted environments and amongst smokers. 

## 4. Material and Methods

All experiments performed in this study were conducted in accordance with our institution’s guidelines and the Declaration of Helsinki.

### 4.1. Cell Culture

Four different strains of human diploid dermal fibroblasts from skin biopsies of healthy women (mastectomy) were used (age 18–38 years). Cells were cultured in Dulbecco’s modified Eagle’s Medium (DMEM; Wisent Inc., Saint-Jean-Baptiste, QC, Canada) supplemented with 5% foetal bovine serum (FBS) (Sigma-Aldrich, Oakville, ON, Canada) and 1% penicillin/streptomycin (Wisent Inc., Saint-Jean-Baptiste, QC, Canada) at 37 °C, 5% CO_2_. 

### 4.2. BaP and Light Exposure

Confluent cell monolayers were pre-treated with BaP (0–500 nM) diluted in phosphate-buffered saline (PBS) for 30 min in dark and then exposed to light. Stock solution of BaP (Sigma-Aldrich, Oakville, ON, Canada) was dissolved in dimethyl sulfoxide (DMSO).

**Figure 5:**
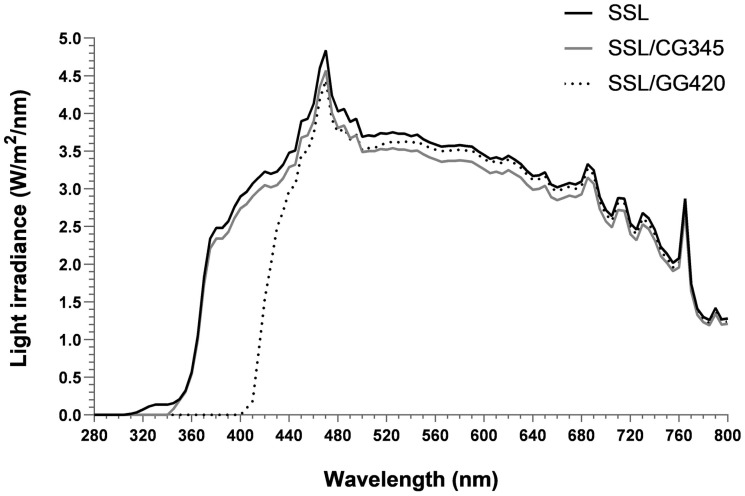
Emission spectra of the SSL with the filters used. Light source consists of an Oriel solar simulator (SSL) with an ozone-free xenon short arc 1.6 kW lamp combined with an air mass 1.5 G (AM1.5 G) filter (black solid line, ssl) and a Schott CGA-345 (grey solid line, UVAssl) or a GG-420 (black dashed line, VLssl) long-pass optical filter.

Irradiations were performed using an Oriel solar simulator (SSL) 1.6 kW with an ozone-free xenon short arc lamp and equipped with a 1.5 G filter ( Newport Corporation, Irvine, CA, USA). For the UVAssl irradiation (>340 nm), UVB and UVA2 were blocked from the SSL spectrum using a CGA-345 filter (Schott, Lebanon, PA, USA). For the VLssl (>400 nm), UVB and UVA were blocked using a GG-420 filter (Schott, Lebanon, PA, USA). Spectra are depicted in Figure 5. UVA irradiance (approximately 2 mW/cm^2^ at cell surface) was measured prior to each irradiation using a UVP UVX Radiometer (Thermo-Fisher, Mississauga, ON, Canada). For VLssl irradiation, the UVAssl corresponding to irradiation time was used. Unirradiated cells were kept in the dark at room temperature. After irradiation, PBS +/− pollutants was replaced with DMEM and cells were incubated at 37 °C, 5% CO_2_, until harvested for analysis. 

### 4.3. Viability Assessment

The viability of skin fibroblasts treated with BaP (0 to 50 nM) and/or irradiated to UVAssl or VLssl (0 to 75 kJ/m^2^) was assessed using an MTS cell viability assay (CellTiter 96^®^ AQueous Non-Radioactive Cell Proliferation Assay; Promega, Madison, WI, USA) 24 h post-exposure, according to the manufacturer’s protocol, as previously performed [6]. The colorimetric analysis was realised using a microplate reader (BioRad 550 Microplate Reader; Bio-Rad, Mississauga, ON, Canada). Values were corrected with background absorbance and the average of the untreated controls was used as a baseline. 

### 4.4. ROS Formation Analysis

Total ROS was measured using the oxidant-sensitive fluorescent probe chloromethyl-2′,7′- dichlorodihydrofluorescein diacetate, acetyl ester (CM-H_2_DCFDA; Thermo-Fisher, Mississauga, ON, Canada), following UVAssl/VLssl and BaP co-exposure. Four different strains of dermal fibroblasts were pre-treated with BaP (0 to 50 nM) and then exposed to 0, 15 or 30 kJ/m^2^ UVAssl or 0, 100 or 200 kJ/m^2^ VLssl. After irradiation, 5µM of CM-H2DCFDA in PBS was added to cells for 60 min in the dark. Fluorescence was measured using a CytoFluor^®^ Series 4000 multi-well fluorescence plate reader (Applied Biosystems, Woburn, MA, USA) (Ex: 480 ± 10/Em: 530 ± 12.5 nm).

### 4.5. Antioxidant Treatments

Cells were treated with 100 µM of α-tocopherol (vitamin E; Sigma-Aldrich, Oakville, ON, Canada) (α-toco) or 100 µM of trolox (Abcam, Cambridge, UK) for 16 h before exposure to BaP, during light exposure, and then medium after irradiation. 

### 4.6. Lipid Peroxidation and MDA Evaluation

Lipid peroxidation was assessed using a lipophilic fluorescent dye sensitive to oxidation, the Bodipy 665/676 (BODIPY™ 665/676, ThermoFisher, Mississauga, ON, Canada). Four strains of dermal fibroblasts, with or without antioxidants pre-treatment, were incubated with BaP (500 nM) in PBS for 30 min and then exposed to 25 kJ/m^2^ UVAssl. After irradiation, PBS was replaced with DMEM-containing antioxidants. After 2 h incubation at 37 °C, 5% CO_2_, cells were stained in a solution of 10 µM of Bodipy diluted in PBS for 30 min at 37 °C, 5% CO_2_. Image acquisition was realised using a Zeiss Axioimager Z2 135 fluorescent microscope coupled with a Zeiss AxioCam MRm Rev 3 Monochromatic Digital Camera. The signal produced was quantified using the “Automeasure” module of AxioVision 4.8.2 (Zeiss, Oberkochen, Germany) and was normalized to the untreated control condition for each cell strain. 

For MDA measurement, dermal fibroblasts, pre-treated (or not) with antioxidants, were incubated with BaP (500 nM) in PBS for 30 min and then exposed to 40 kJ/m^2^ UVAssl. After irradiation, PBS was discarded and replaced with PBS containing (or not) antioxidants. After 2 h incubation at 37 °C, 5% CO_2_, cells were harvested and MDA formation was assessed using the lipid peroxidation (MDA) assay kit (Abcam, Cambridge, UK) with some modifications. Briefly, after cell lysis and protein normalization, thiobarbituric acid (TBA) was added to samples during 1 h at 95 °C. MDA production was detected by fluorescence using a CytoFluor^®^ Series 4000 multi-well fluorescence plate reader (Applied Biosystems, Woburn, MA, USA) (Ex: 532 nm/Em: 572 nm). Results were normalized to the untreated control. 

### 4.7. Bulky DNA Adducts’ Formation 

Bulky DNA adducts were detected using a modified large amplicon quantitative PCR (polymerase chain reaction) assay (LA-qPCR) [66,67]. The technique is based on the decrease in amplification efficiency caused by bulky-lesion-induced blockage of DNA polymerase [68]. LA-qPCR was performed according to a protocol modified from Furda et al. (2014) [69], to allow for the simultaneous amplification of large and short targets under the same PCR parameters. Primers for large (4889 bp) and short (66 bp) nuclear targets were designed for the human corin serine peptidase gene (CORIN) using the primer designing tool Primer-BLAST [70].

Primary dermal fibroblasts were treated with BaP (0–200 nM) and exposed to UVAssl (200 kJ/m^2^). After irradiation, cells were incubated for 6 h and DNA was extracted using the DNeasy Blood and Tissue Kit (Qiagen, Toronto, ON, Canada), according to the manufacturer’s protocol. The LA-qPCR was performed on a Rotor-Gene Q real-time thermocycler (Qiagen) using the following protocol: 3 min at 95 °C followed by 70 cycles of 20 s at 95 °C and 40 s at 60 °C. For each sample, the relative amount of bulky DNA adducts is defined as the delay in amplification between large and short amplicons, normalized with the ΔCt of the control condition (No PAHs in dark) and calculated as ΔΔCt = (Ct_sample_ short amplification − Ct_sample_ large amplification)/(Ct_control_ short amplification − Ct_control_ large amplification). 

### 4.8. Statistical Analysis

Statistical analyses were performed using GraphPad Prism version 9 software (GraphPad Software, San Diego, CA, USA). Differences between conditions were assessed using a one-way analysis of variance (ANOVA1) with Tukey HSD procedure as a post hoc test. A *p*-value ≤ 0.05 was defined as statistically significant.

## Data Availability

Dataset available on request from the authors.
